# Microbiological Characteristics of Necrotizing Fasciitis at Sultan Qaboos University Hospital, Muscat, Oman: A Retrospective Study

**DOI:** 10.7759/cureus.104686

**Published:** 2026-03-04

**Authors:** Abeer Altobi, Maryam Albreiki, Abdullah Al Lawati, Asmaa Al Furqani, Moath Shummo, Nawaf A Al-Muqaimi, Thuraya Al Busaidi, Ibrahim Al Busaidi

**Affiliations:** 1 Plastic and Reconstructive Surgery, Sultan Qaboos University Hospital, Muscat, OMN; 2 Hematology, Sultan Qaboos University Hospital, Muscat, OMN; 3 Medicine, Sultan Qaboos University Hospital, Muscat, OMN

**Keywords:** antibiotics, microbiology, mortality, multidrug resistance, necrotizing fasciitis

## Abstract

Introduction

Necrotizing fasciitis is a life-threatening, rapidly progressing infection of the subcutaneous tissue and fascia. Diabetes mellitus, immunosuppression, and other comorbid conditions are primary predisposing factors. The lower limbs are the most commonly affected site. Necrotizing fasciitis is classified into two main microbiological categories: type I (polymicrobial infection), which is more prevalent, and type II (monomicrobial infection). Early clinical recognition, prompt surgical debridement, and timely initiation of appropriate empirical antimicrobial therapy are crucial for effective management.

Methods

A retrospective study was conducted over a five-year period (2015-2021), including adult patients (≥18 years) with a confirmed diagnosis of necrotizing fasciitis who had available clinical data and a follow-up period of 12 months. Clinical parameters, including comorbid conditions, infection sites, and management approaches (surgical and medical), were documented. Additionally, microbiological profiles and antimicrobial resistance patterns were analysed to determine their impact on 30-day mortality.

Results

A total of 43 patients were included, with 31 males (72%) and 12 females (28%), and a median age of 61 years (range: 18-85 years). Diabetes mellitus was the most prevalent comorbid condition, affecting 31 patients (72%). Lower limbs were involved in 31 cases (72%). Type I and type II infections were nearly equally distributed, accounting for 22 cases (51%) and 21 cases (49%), respectively. The predominant isolated organisms were *Pseudomonas aeruginosa* in four cases (9%), *Escherichia coli* in four cases (9%), *Klebsiella pneumoniae* in three cases (7%), methicillin-resistant *Staphylococcus aureus* (MRSA) in three cases (7%), and *Proteus mirabilis* in three cases (7%). Extended-spectrum β-lactamase-producing organisms were identified in two cases (5%). Multidrug-resistant (MDR) *Pseudomonas aeruginosa* was found in four cases (9%), MDR *Klebsiella pneumoniae* in three cases (7%), and MDR *Acinetobacter *species in two cases (5%). The total prevalence of MDR organisms was eight cases (19%). The 30-day mortality rate was recorded in three patients (7%).

Conclusion

Necrotizing fasciitis was more prevalent among males, with diabetes mellitus identified as the leading predisposing factor. The lower limbs were the most commonly affected site, with nearly equal distribution of type I and type II infections. Gram-negative and MDR organisms were common, though MDR infections were not significantly associated with mortality. Early diagnosis, timely surgical intervention, and targeted antimicrobial therapy likely contributed to the low mortality rate (7%). Emphasising the need for regional-specific resistance patterns and antimicrobial strategies.

## Introduction

Necrotizing fasciitis (NF), commonly known as a flesh-eating disease, is a rare but aggressive infection affecting the superficial fascia and subcutaneous tissues [1]. It is associated with rapid tissue necrosis, systemic toxicity, and high mortality rates [2]. NF can affect many parts of the body; however, the lower limbs are the most frequently involved sites. Risk factors include diabetes mellitus, chronic kidney disease, liver cirrhosis, hypertension, immunosuppression, and malignancy [3].

NF can be classified into two main microbiological categories: type I (polymicrobial) and type II (monomicrobial). Type I infections involve anaerobic bacteria and facultative anaerobes, including *Enterobacteriaceae* and *Bacteroides* species, often seen in perineal and postoperative infections [4]. Type II infections, commonly caused by Group A *Streptococcus* and *Staphylococcus aureus*, are more prevalent in healthy individuals [2].

Early diagnosis is crucial, relying mainly on clinical presentation, laboratory parameters and imaging modalities are useful to support the diagnosis [4]. There are classic manifestations of NF that patients usually present with, such as erythema, severe pain, fever and oedema. Classically, these manifestations progress in severity very rapidly, which makes NF distinguishable clinically. Wong et al. developed the Laboratory Risk Indicator for Necrotizing Fasciitis (LRINEC) score, which uses markers and biochemical parameters to stratify NF risk [5]. The cornerstone of NF uses inflammatory management includes surgical debridement, broad-spectrum antimicrobial therapy, aggressive resuscitation, and supportive care [6]. Timely surgical debridement is found to be the most important lifesaving intervention. Also, given the increasing prevalence of MDR pathogens, empirical antibiotic regimens should be tailored based on local microbiological data [7].

This study aims to characterize the microbiological profile and antimicrobial resistance patterns of adult patients with NF admitted to Sultan Qaboos University Hospital (SQUH), Oman. The primary outcome was 30-day mortality. Secondary objectives included evaluation of multidrug-resistant (MDR) organisms and statistical assessment of the association between MDR status and mortality. Given the limited regional data on NF, this study seeks to provide structured epidemiological and outcome-based insights within the local healthcare setting.

## Materials and methods

Study design and setting

A retrospective observational study was conducted at SQUH, a tertiary care hospital in Oman, to evaluate the microbiological and clinical characteristics of NF. Records of patients who presented to SQUH, whether as inpatients or outpatients, between January 2015 and January 2021 were reviewed.

Study population and inclusion/exclusion criteria

All adult patients (≥18 years) with a confirmed diagnosis of NF between January 2015 and January 2021 were included. NF was confirmed based on clinical presentation consistent with rapidly progressive soft tissue infection (e.g., severe pain out of proportion, erythema, oedema, systemic toxicity) combined with intraoperative findings of fascial necrosis and/or histopathological confirmation when available. Imaging studies (e.g., CT or MRI) were considered supportive but were not used as the sole diagnostic criterion. Only patients with available microbiological culture data were included.

Sample size

All NF patients who satisfied the inclusion criteria during the study period were included. A formal sample size calculation was not required, as all eligible cases were reviewed. 

Data collection

Clinical data, including demographics, comorbid conditions, infection sites, microbiological findings, antimicrobial resistance and 30-day mortality, were extracted from the SQUH electronic medical records (Track Care System). An immunocompromised state was defined as documented malignancy, chronic corticosteroid use, organ transplantation, advanced renal disease or other conditions associated with impaired immune function as recorded in the medical record. A standardized data collection sheet was designed based on commonly reported variables in the literature and reviewed by consultant supervisors in Plastic Surgery and Infectious Diseases.

Microbiological analysis was based primarily on intraoperative tissue cultures; wound swabs were included when tissue samples were unavailable. Antimicrobial susceptibility testing followed Clinical and Laboratory Standards Institute (CLSI) guidelines. Multidrug resistance (MDR) was defined as resistance to three or more antimicrobial classes. Infections were classified as polymicrobial (type I) or monomicrobial (type II) based on the number of organisms isolated.

Statistical analysis

Data were analysed using SPSS (IBM SPSS Statistics for Windows, IBM Corp., Version 27, Armonk, NY), employing descriptive and inferential statistical methods. Categorical variables were expressed as frequencies and percentages, while continuous variables were summarized using median and range. Chi-square tests were used to assess associations between categorical variables, particularly between microbiological characteristics and clinical outcomes, with a p-value < 0.05 considered statistically significant. Cases with incomplete microbiological data were excluded at the selection stage, and no statistical imputation was performed; analyses were conducted using available data only.

Ethical consideration

Institutional Review Board (IRB) approval was obtained by the Medical Research Ethics Committee (MREC) at the College of Medicine and Health Sciences, Sultan Qaboos University (approval number: #2568). The study adhered to all the principles of the Declaration of Helsinki.

## Results

Demographic and clinical characteristics

**Figure 1:**
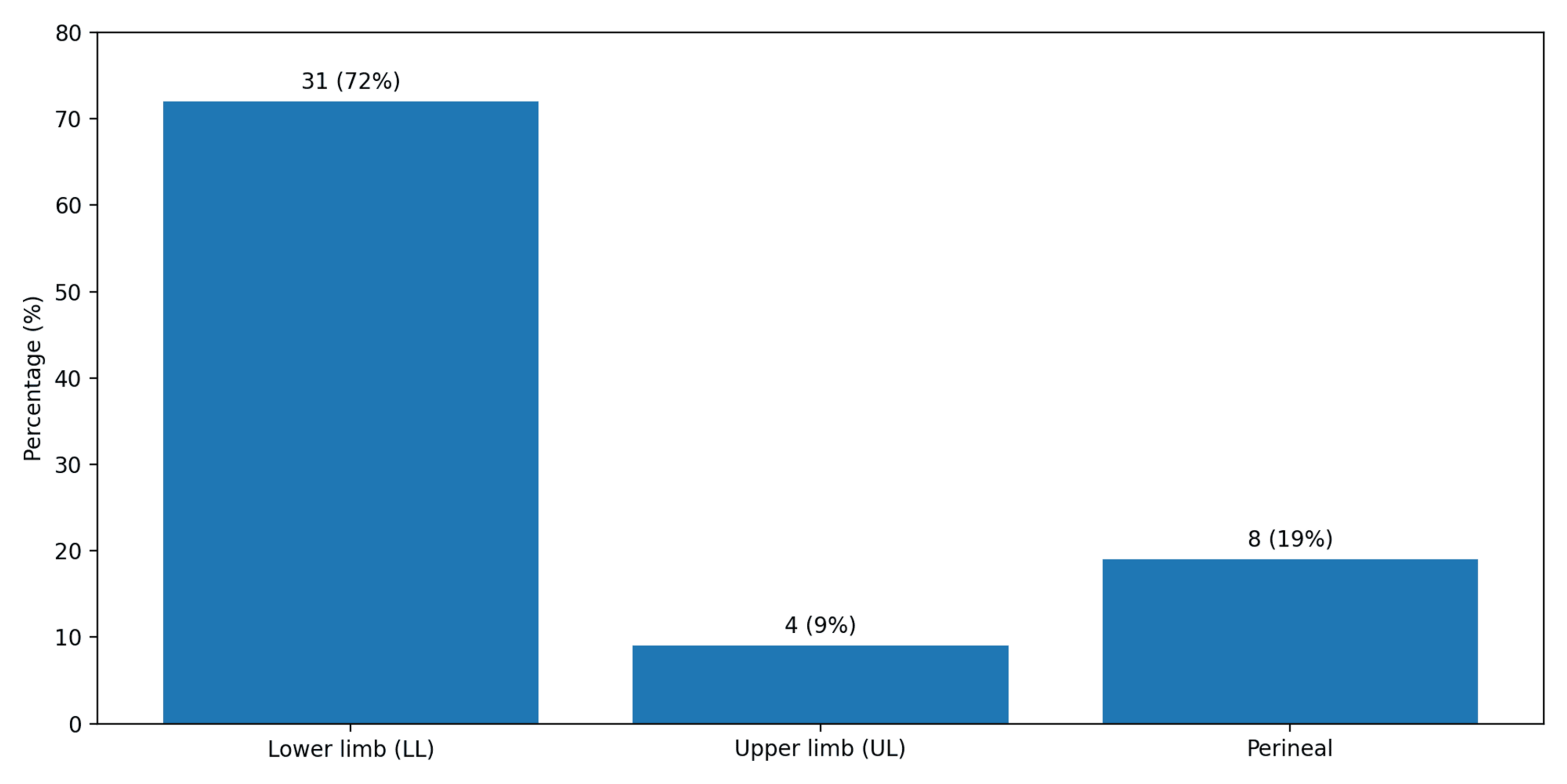
Anatomical sites involved in necrotizing fasciitis Values are expressed as absolute numbers with corresponding percentages (n, %).

As shown in Table 1, of the 43 patients included, 31 were male (72%). The median age of the included group was 61 years (range: 18-85 years). The most prevalent comorbidity was diabetes mellitus, affecting 31 patients (72%), followed by end-stage renal disease in 10 patients (23%) and immunosuppression in 3 patients (7%). The lower limbs were the most affected site, accounting for 31 cases (72%), followed by the perineum in eight cases (19%) and the upper limbs in four cases (9%), as shown in Figure 1.

**Table 1:** Demographic and baseline characteristics of the study population (n = 43)

Characteristic	Value
Age (years)	
Mean ± SD	60.7 ± 15.1
Median (range)	61 (18-85)
Sex, n (%)	
Male	31 (72%)
Female	12 (28%)
Comorbidities, n (%)	
Diabetes mellitus	31 (72%)
Peripheral vascular disease	2 (5%)
End-stage renal disease	10 (23%)
Immunocompromised state	3 (7%)

Microbiological findings and antimicrobial resistance

**Figure 2:**
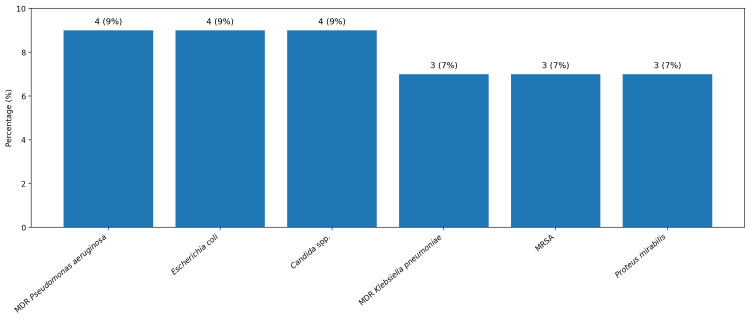
Prevalence of commonly isolated organisms in necrotizing fasciitis cases Values are expressed as absolute numbers with corresponding percentages (n, %).

Among NF cases, 22 cases (51%) were polymicrobial (type I), while 21 cases (49%) were monomicrobial (type II). *Pseudomonas aeruginosa* and *Escherichia coli* were the most frequently isolated organisms, identified in four cases each (9%), as shown in Figure 2. *Klebsiella pneumoniae*, MRSA, and *Proteus mirabilis* were each isolated in three cases (7%). Extended-spectrum β-lactamase-producing organisms were identified in two cases (5%). MDR *Pseudomonas aeruginosa* was detected in four cases (9%), MDR *Klebsiella pneumoniae* in three cases (7%), and MDR *Acinetobacter* species in two cases (5%). The overall prevalence of MDR organisms was eight cases (19%).

30-day mortality and MDR associations

The overall 30-day mortality rate was three cases (7%). No statistically significant association was found between MDR infections and mortality (p = 0.275).

## Discussion

This study provides valuable insight into the clinical and microbiological profile of necrotising fasciitis (NF) in a tertiary care setting in Oman. The observed male predominance (72%) and association with diabetes mellitus (DM) (72%) align with the global epidemiological trends reported in multiple studies [2,7-10]. DM is a well-established risk factor for NF due to its role in impairing immune function, causing vascular insufficiency, and contributing to poor wound healing [9,11]. These findings are consistent with previous studies reporting DM prevalence ranging from 40% to 70% among NF patients, confirming its role as the most important predisposing factor worldwide [7,9,12].

The high prevalence of lower limb involvement in this study is also consistent with prior reports identifying the lower extremities as the most frequently affected site, particularly in diabetic populations [2,9,13]. This vulnerability is attributed to peripheral neuropathy, vascular insufficiency, and increased trauma susceptibility [11,13]. Recent multicentre studies have further emphasized that lower limb NF is associated with delayed presentation and an increased risk of complications, reinforcing the importance of early detection in high-risk populations [14].

Notably, a near-equal distribution of type I (polymicrobial, 51%) and type II (monomicrobial, 49%) NF was observed in this study. Traditionally, polymicrobial infections account for approximately 70-80% of NF cases [2,6,9]. However, recent epidemiological shifts have shown increasing variation in microbiological patterns with higher rates of monomicrobial infections reported in certain regions [10,15]. This variation may reflect regional microbiological ecology, differences in health care exposure, and underlying patient comorbidities. These findings highlight the importance of regional epidemiological surveillance to guide effective empirical antimicrobial therapy.

The microbiological profile observed in this study showed a predominance of gram-negative organisms, including *Pseudomonas aeruginosa*, *Escherichia coli*, and *Klebsiella pneumoniae*, as well as methicillin-resistant *Staphylococcus aureus* (MRSA). While historically group A *Streptococcus* was considered the most common pathogen in monomicrobial NF, recent studies have reported an increasing role of gram-negative organisms, particularly in healthcare-associated infections and among immunocompromised patients [2,9,16]. Similar trends have been observed in recent global and regional studies, where gram-negative pathogens and mixed infections are increasingly reported, especially in diabetic and hospitalized populations [14,16,17].

The prevalence of MDR organisms in this study (19%) is consistent with growing global concerns about antimicrobial resistance in severe soft-tissue infections. Recent international surveillance studies have reported MDR prevalence rates ranging from 15% to 35% in NF cases, particularly involving *Pseudomonas aeruginosa*, *Acinetobacter baumannii* and ESBL-producing *Enterobacteriaceae* [17,18]. The emergence of MDR pathogens poses significant challenges in selecting appropriate empirical therapy and highlights the importance of local antibiograms in guiding treatment decisions.

Despite the presence of MDR organisms, no statistically significant association with mortality rate was observed. This aligns with previous studies demonstrating that early surgical debridement remains the most important determinant of survival, outweighing microbiological factors alone [19,20]. Prompt and aggressive surgical debridement reduces bacterial load, limits toxin production and prevents systemic progression, significantly improving patient outcomes regardless of the infecting organism [19]. Several large retrospective studies have demonstrated that delayed surgical intervention is one of the strongest predictors of mortality in NF [13,20].

The low mortality rate (7%) observed in this study was lower than the 20-30% mortality reported in most international studies [2,9,10,19]. This favourable outcome likely reflects early diagnosis, prompt surgical intervention, appropriate antimicrobial therapy and multidisciplinary care in a tertiary healthcare setting. Recent studies have demonstrated that early aggressive management, particularly surgical intervention within the first 24 hours, significantly reduces mortality rates [13,14,20].

Limitations

The low mortality rate, while encouraging, warrants cautious interpretation. Selection bias may exist, as patients without microbiological data (potentially more critically ill or rapidly fatal cases) were excluded. Furthermore, the single-centre design and small sample size (n = 43) limit generalizability. Although early surgical intervention is widely supported in the literature as a determinant of improved outcomes, its direct impact was not quantitatively analysed in this cohort. The low number of mortality events (n = 3) limits statistical power, and the absence of statistical significance should not be interpreted as the absence of effect. Additionally, no multivariate analysis was performed to adjust for potential confounders, limiting causal interpretation.

## Conclusions

NF remains a life-threatening infection with significant morbidity. This study highlights its male predominance, strong association with diabetes mellitus and near-equal distribution of type I and type II infections. The frequent isolation of Gram-negative and MDR organisms, particularly *Pseudomonas aeruginosa* and *Escherichia coli*, reflects evolving microbiological patterns. Despite MDR pathogens being present in some of the cases, no statistically significant association with mortality was detected. The relatively low 30-day mortality rate underscores the importance of early diagnosis, aggressive surgical management and tailored antimicrobial coverage. These findings should be interpreted within the constraints of the study’s small sample size and retrospective design.
